# Retrospective Evaluation of the Prognostic Value of Histological Growth Pattern in Patients with Colorectal Peritoneal Metastases Undergoing Curative-Intent Cytoreductive Surgery

**DOI:** 10.1245/s10434-024-15125-y

**Published:** 2024-03-15

**Authors:** Leonel Kamdem, Antoine El Asmar, Pieter Demetter, Ismael Coulibaly Zana, Charif Khaled, Francesco Sclafani, Vincent Donckier, Peter Vermeulen, Gabriel Liberale

**Affiliations:** 1 Department of Surgery, Institut Jules Bordet, Université Libre de Bruxelles, Brussels, Belgium; 2 Department of Pathology, Institut Jules Bordet, Université Libre de Bruxelles, Brussels, Belgium; 3 Department of Medical Oncology, Institut Jules Bordet, Université Libre de Bruxelles, Brussels, Belgium; 4 Translational Cancer Research Unit, Department of Oncological Research, Oncology Center GZA, GZA Hospitals St. Augustinus, University of Antwerp, Antwerp, Belgium

**Keywords:** Histological growth patterns, Peritoneal carcinomatosis, Colorectal cancer, Cytoreductive surgery, HIPEC

## Abstract

**Background:**

Two distinct histological growth patterns (HGPs) were described in patients with peritoneal metastasis of colorectal cancer origin (PMCRC) with limited Peritoneal Cancer Index (PCI) ≤ 6 who did not receive neoadjuvant chemotherapy (NAC) and were treated with cytoreductive surgery (CRS) ± hyperthermic intraperitoneal chemotherapy (HIPEC): pushing HGP (P-HGP) and infiltrating HGP (I-HGP). Patients with dominant P-HGP (> 50%) had significantly better disease-free survival (DFS) and overall survival (OS).

**Objective:**

We aimed to determine whether these previous observations regarding the prognostic value of HGP in patients with PMCRC with low PCI (≤ 6) are also valid in all operable patients, regardless of whether they received NAC or not and regardless of PCI score.

**Methods:**

This was a retrospective study including 76 patients who underwent complete CRS ± HIPEC for PMCRC between July 2012 and March 2019. In each patient, up to five of the largest excised peritoneal nodules were analyzed for their tumor-to-peritoneum interface. Correlations between NAC, HGP, and prognosis were further explored.

**Results:**

Thirty-seven patients (49%) had dominant P-HGP and 39 (51%) had dominant I-HGP. On univariate analysis, patients with P-HGP ≤ 50% had significantly lower OS than those with dominant P-HGP > 50% (39 versus 60 months; *p *= 0.014) confirmed on multivariate analysis (hazard ratio 2.4, 95% confidence interval 1.3–4.5; *p *= 0.006). There were no significant associations between NAC and type of HGP.

**Conclusions:**

This study confirms the prognostic value and reproducibility of the two previously reported HGPs in PMCRC. Dominant P-HGP is associated with better DFS and OS in patients undergoing curative-intent CRS ± HIPEC compared with I-HGP, independently of the extent of peritoneal disease burden.

Colorectal cancer (CRC) represents the third most commonly diagnosed cancer worldwide and the second cause of cancer-associated mortality.^1^ An estimated 15% of these patients present with synchronous liver metastases (LM) at diagnosis^2,3^ and 7% present with peritoneal metastases (PM).^4^ Furthermore, an additional 16–20% will develop metachronous LM within 3 years after diagnosis, and up to 19% will develop PM, even after curative-intent surgery.^2,4,5^

In patients with limited PM, cytoreductive surgery (CRS) with or without hyperthermic intraperitoneal chemotherapy (HIPEC), associated or not with perioperative systemic chemotherapy, is an established therapeutic option with a 5-year survival rate of up to 30% when CRS is complete.^6^ The most important prognostic factors used to define patient eligibility for surgical management are the extent of the peritoneal disease, determined by the Peritoneal Cancer Index (PCI), and the completeness of CRS.^6^ In addition, effort has been made to identify other prognostic factors in patients with PM of CRC origin (PMCRC), including clinical biomarkers and the molecular biology of the tumor.^7,8^

More recently, histological growth pattern (HGP) has been reported to be a major prognostic factor in patients undergoing colorectal LM resection. By analyzing the hematoxylin and eosin (H&E)-stained slides and the slides showing the reticulin pattern of the LM, three different growth patterns were described: a desmoplastic growth pattern where the metastases are separated from the surrounding liver parenchyma by a rim of desmoplastic stroma in which a dense lymphocytic infiltrate is present; a pushing growth pattern where at the tumor–liver parenchyma interface, the liver plates are pushed aside and run in parallel with the circumference of the metastases from which they are separated by a thin layer of reticulin fibers without desmoplastic stroma; and a replacement growth pattern where tumor cells are replacing hepatocytes in the liver plates, conserving the reticulin network of the liver parenchyma.^9–17^ Similarly, by studying the interactions between colorectal PM and the peritoneum, we have recently described two distinct HGPs in patients with limited colorectal peritoneal disease (PCI ≤ 6) treated with CRS ± HIPEC: the pushing-HGP (P-HGP), where the healthy tissue is pushed back by a fibrous rim, with absence of focal penetration of tumor cells into the surrounding peritoneal lining (Fig. 1a); and the infiltrating-HGP (I-HGP), where we observed focal penetration of tumor cells into the surrounding peritoneal lining, without a separating fibrous rim (Fig. 1b).^18^ The dominant P-HGP was associated with a more favorable prognosis in patients with PMCRC compared with I-HGP, with median disease-free survival (DFS) of 30 versus 9 months and a median overall survival (OS) of 131 versus 41 months, respectively. Despite these promising results, the study was based on selected patients with limited peritoneal disease (PCI ≤ 6) who did not receive any neoadjuvant chemotherapy (NAC) prior to CRS ± HIPEC. In patients with LM of CRC origin (LMCRC), it has been reported that NAC favors an increase in the prevalence of the desmoplastic HGP.^19^ In PMCRC, the prognostic role of HGP in patients with a PCI >6 and/or who received NAC is yet to be determined.

**Fig. 1:**
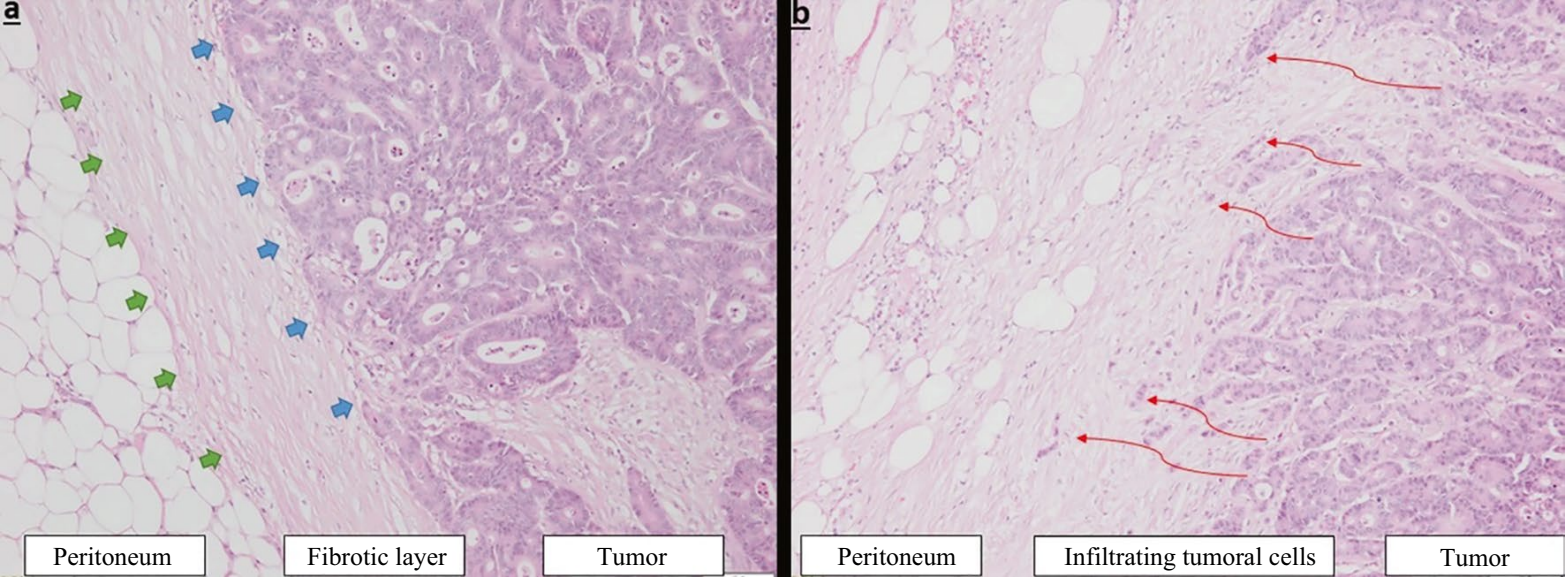
Tumor-peritoneum histological growth pattern observed in peritoneal metastasis of colorectal cancer origin. **a** Pushing HGP in PMCRC. **b** Infiltrating HGP in PMCRC. *HGP* histological growth pattern, *PMCRC* peritoneal metastasis of colorectal cancer origin. *Note* Image reproduced without change from a previously published work by our department and in the same journal, *Annals of Surgical Oncology* (El Asmar A, Demetter P, Fares F, Sclafani F, Hendlisz A, Donckier V, et al. The Prognostic Value of Distinct Histological Growth Patterns of Colorectal Peritoneal Metastases: A Pilot Study. Ann Surg Oncol. 2023 Jun;30(6):3320-3328), abiding with the creative common license (https://s100.copyright.com/AppDispatchServlet?title=The%20Prognostic%20Value%20of%20Distinct%20Histological%20Growth%20Patterns%20of%20Colorectal%20Peritoneal%20Metastases%3A%20A%20Pilot%20Study&author=Antoine%20El%20Asmar%20MD%20et%20al&contentID=10.1245%2Fs10434-023-13118-x&copyright=The%20Author%28s%29&publication=1068-9265&publicationDate=2023-02-08&publisherName=SpringerNature&orderBeanReset=true&oa=CC%20BY)

The objective of this study was to determine whether the previously described HGPs in PMCRC are observed in all patients undergoing CRS, with or without NAC, with or without HIPEC, and no matter what their PCI. Moreover, we aimed to evaluate the prognostic impact of these HGPs in patients with PMCRC.

## Materials and Methods

### Design and Inclusion Criteria

We retrospectively reviewed all patients who underwent CRS with curative intent (R0/R1 resection) ± HIPEC (intraperitoneal oxaliplatin 460 mg/m^2^ at 41–43 °C for 30 min, and systemic 5-fluorouracil) for PMCRC at our institution between July 2012 and March 2019. Patients who had incomplete macroscopic CRS were excluded.

Most patients were evaluated in the 6 weeks prior to surgery by morphologic imaging, including abdominopelvic computed tomography (CT) and/or magnetic resonance imaging (MRI), and in the case of suspected lesion and/or high risk of metastatic dissemination, by fluorodeoxyglucose positron emission tomography (FDG-PET) scan.

Each patient was discussed several times, and individually, at the weekly digestive oncologic multidisciplinary meeting, prior to the beginning of their treatment to decide on the best treatment plan, and during their treatment to evaluate their response and imaging/pathological response. In that setting, and in compliance with the latest literature research, for each patient it was decided whether they would undergo CRS with NAC, HIPEC, or adjuvant chemotherapy, or a combination of any of the three chemotherapy options mentioned.

This study was approved by the Institut Jules Bordet (ULB) Ethics Committee (CE3222).

### Pathology

All pathology reports from the operative specimens of the patients were reviewed. For each patient, up to five nodules were chosen, including the largest nodule, the nodule in direct contact with the peritoneum, and the nodule in which the whole circumferential tumor/peritoneal interface could be assessed. The corresponding pathology slides were analyzed for their HGP by three pathologists (PV, ICZ, and PD). Slides showing the largest circumferential margins between the tumor and the peritoneal/subperitoneal tissues were included. Peritoneal nodules confined within a resected organ (e.g., spleen, liver, and ovary) were excluded. The tumor periphery was assessed for HGP on H&E-stained slides. Cases where the PM consisted of only fibrotic and/or necrotic tissue, or when the tumor-peritoneum interface could not be analyzed, were considered non-assessable (NA) and were excluded from further analysis. HGP evaluation was performed using a Leica bright-field microscope at a low magnification (10 × objective). For each slide, the relative presence (expressed as a percentage) of the different HGPs at the tumor-peritoneal interface was estimated. The relative proportion of each HGP from the total interface length was then calculated for each nodule. A particular growth pattern was considered dominant whenever it demarcated ≥ 50% of the nodule-peritoneum interface, as per the international consensus guidelines for scoring the histological growth patterns of LM,^20^ as there is not yet any consensus on PM. The mean HGP scores were calculated for the selected nodules from each patient.

### Statistical Analysis

Clinical data included patient demographics, primary tumor histological and molecular features, type of NAC (if any), DFS, and OS. These were pseudonymized and merged into a study database using REDCap and the statistical analyses were performed using SAS version 9.4 (SAS Institute Inc., Cary, NC, USA). The associations between the included variables and OS and DFS were evaluated using the Kaplan–Meier (KM) test and the Cox regression model. OS was defined as the time interval between the date of CRS and the date of death from any cause, or the last follow-up. DFS was defined as the time interval between the date of CRS and the date of first recurrence or death. Patients who were lost to follow-up were censored at the date they were known to be alive and disease-free. Univariate analysis was performed to evaluate prognostic factors using the KM method, and the log-rank test was used to calculate and compare the survival curves for DFS and OS in patients with different HGPs. Multivariate Cox regression analysis was performed to adjust for potential confounding factors. Covariates introduced into the multivariate models were the statistically significant variables and age. The Chi-square test was used to evaluate the associations between NAC and the prevalence of HGPs. To do this, the distribution of HGPs among chemo-naive and NAC-treated patients was compared. Patients who had received any chemotherapy after the diagnosis of PM prior to CRS were considered NAC-treated.

## Results

### Patients

We identified 94 patients who underwent complete CRS ± HIPEC for PMCRC. Among these, 18 patients were excluded: 10 had no available archived slides for analysis, 5 had intraovarian metastases only, and 3 had intra-splenic metastases only, resulting in 76 patients included in our study.

Patient characteristics are reported in Table 1. The study included 29 males and 47 females, with a median age of 59 years. Primary tumors were of colonic origin in 89% of patients and of rectal origin in 11% of patients. Twenty-six patients (34%) received NAC and the median PCI for the whole population was 6.

**Table 1:** Univariate analysis of clinical, demographic, and histological variables on outcomes

Variable	*N* (%)	DFS		OS	
		Median DFS (range, in months)	*p*-Value	Median OS (range, in months)	*p-*Value
Age, years	76 (100)	9 (6.7–11.6)	0.488	50 (35.4–67.8)	0.127
>59	38 (50)	9 (6.7–15.8)		55 (38–91.5)	
≤59	38 (50)	8 (5.7–11.6)		35 (23.4–54.8)	
Sex	76 (100)	9 (6.7–11.6)	0.952	50 (35.4–67.8)	0.874
Male	29 (38.2)	10 (6.4–14.8)		52 (35.4–71.4)	
Female	47 (61.8)	7 (5.7–9.7)		43 (26.5–69.8)	
MMR	47 (100)	10 (6.7–14.8)	**0.021**	51 (35.7–67.8)	**0.049**
MSS	41 (87.2)	9 (6.6–9.9)		43 (35.4–55.6)	
MSI	6 (12.8)	–		–	
KRAS	57 (100)	9 (6.7–11.2)	0.351	49 (35.4–59.6)	0.623
Wild-type	30 (52.6)	9 (6.3–15.8)		38 (25.8–59.6)	
Mutated	27 (47.4)	8 (6–12)		52 (38.7–69.8)	
Tumor grade differentiation	76 (100)	9 (6.7–11.6)	0.886	50 (35.4–67.8)	0.851
Well	23 (30.3)	9 (5.6–11.8)		41 (25.8–67.8)	
Moderately	37 (48.7)	10 (6.4–14.8)		52 (37.9–71.4)	
Poorly	16 (21)	7 (2.1–42.6)		21 (13.2–NA)	
Lymph node status	76 (100)	9 (6.7–11.6)	**0.008**	50 (35.4–67.8)	**0.021**
pN0	20 (26.3)	13 (6.7–NA)		91 (35.4–NA)	
pN1	28 (36.8)	9 (5.6–15.9)		50 (26.5–71.5)	
pN2	28 (36.8)	6 (3.9–9.7)		36 (21.3–51.7)	
pT^a^	75 (100)	9 (6.7–11.6)	0.827	50 (35.7–67.8)	0.952
1	1 (1)	7 (NA)		60 (NA)	
2	2 (3)	20 (NA)		NA	
3	33 (44)	8 (3.9–15.7)		49 (27.1–75.5)	
4	39 (52)	9 (6.4–11.8)		51 (26.5–71.4)	
Localization	76 (100)	9 (6.7–11.6)	0.535	50 (35.4–67.8)	0.881
Colon	68 (89)	9 (6.7–11.8)		51 (35.4–69.8)	
Rectum	8 (11)	6.7 (3–19.8)		38 (21.3–NA)	
Peritoneal metastases	76 (100)	9 (6.7–11.6)	0.880	50 (35.4–67.8)	0.995
Metachronous	46 (61)	10 (6–15.7)		52 (35.7–67.8)	
Synchronous	30 (39)	8 (6.3–12)		38 (23.4–85.6)	
ASA score	73 (100)	9 (6.7–11.6)	0.350	51 (35.4–67.8)	0.907
2	55 (75)	8 (6.3–13.4)		51 (33.6–69.8)	
3	18 (25)	9 (6–14.8)		43 (27.1–NA)	
CEA	71 (100)	9 (6.6–11.2)	0.512	51 (35.4–67.8)	0.907
≤5	35 (49)	9 (6.4–14.8)		38 (26–85.6)	
>5	36 (51)	7 (5.8–11.6)		51 (35.7–59.6)	
CA19.9	40 (100)	9 (6.6–12)	0.623	38 (24–69.8)	0.712
≤18	20 (50)	11 (3.9–18.9)		31 (18.6–91.5)	
>18	20 (50)	7 (3.3–12)		47 (24–71.5)	
PCI	76 (100)	9 (6.7–11.6)	**0.001**	50 (35.4–67.8)	**0.007**
≤6	41 (54)	13 (7.8–19.8)		72 (38.7–132)	
>6	35 (46)	6 (3.3–9.7)		35 (20.1–52.1)	
Liver metastasis	76 (100)	9 (6.7–11.6)	**0.001**	50 (35.4–67.8)	**0.001**
No	40 (53)	13 (7.1–19.8)		71 (48.8–NA)	
Yes	36 (47)	7 (5.3–8.5)		34 (19.6–43.4)	
Neoadjuvant chemotherapy before CRS	76 (100)	9 (6.7–11.6)	**0.035**	50 (35.4–67.8)	0.295
No	50 (66)	10 (7–15.8)		55 (33.6–71.4)	
Yes	26 (34)	7 (5.6–8.5)		38 (20.9–59.6)	
Size of nodules, mm	68 (100)	9 (6.6–11.6)	0.798	52 (35.7–69.8)	0.163
≤26	34 (50)	9 (5.8–11.8)		60 (35.4–NA)	
>26	34 (50)	8 (6–14.8)		42 (28.8–55.6)	
HGP	76 (100)	9 (6.7–11.6)	0.091	50 (35.4–67.8)	**0.014**
P-HGP ≤50%	39 (51)	8 (5.7–9.7)		39 (26.2–51.7)	
P-HGP >50%	37 (49)	12 (6.3–18.2)		60 (35.4–NA)	

Bold values indicate statistically significant results (*p* ≤ 0.05)

^*^None of the patients in this series had pT1-2 primary tumors or a pre-operative ASA 1 or 4 score

*ASA* American Society of Anesthesiologists, *CA19-9* carbohydrate antigen 19-9, *CEA* carcinoembryonic antigen, *CRS* cytoreductive surgery, *DFS* disease-free survival, *HGP* histological growth pattern, *NA* not available, *MMR* mismatch repair, *MSI* microsatellite instability, *MSS* microsatellite stable, *OS* overall survival, *PCI* Peritoneal Cancer Index, *P-HGP* pushing-HGP

### Histological Growth Pattern

In total, 206 PM nodules were analyzed for HGP in 76 patients. The median number of PM nodules analyzed per patient was two, with a median size of 26 mm. We observed the same two previously described HGPs: the pushing-HGP (P-HGP) and the infiltrating-HGP (I-HGP).^18^ A dominant P-HGP was found in 37 (49%) patients, and a dominant I-HGP was found in 39 (51%) patients. Almost no discordance was found between the readings of pathologists. Furthermore, little to no difference was found in HGP percentage between nodules from the same patient.

### Prognostic Factors for Disease-Free Survival and Overall Survival

The univariate analysis of prognostic factors for DFS and OS are reported in Table 1. A lower PCI (≤ 6), absence of locoregional lymph node (pN0) involvement, absence of synchronous LM, and microsatellite instability (MSI) status were favorable prognostic factors for DFS and OS. Furthermore, NAC prior to CRS was a predictor of better DFS, while a dominant P-HGP was a predictor of better OS.

Moreover, on multivariate analysis, P-HGP ≤ 50%, PCI ≤ 6, and the presence of LM were significantly associated with OS and DFS.

### Prognostic Role of Histological Growth Pattern (HGP)

In terms of OS, patients with P-HGP > 50% had a better prognosis than those with a dominant I-HGP: 60 versus 39 months, respectively (*p *= 0.014)*.* In terms of DFS, patients with a dominant P-HGP still showed a more favorable prognosis (12 versus 8 months, respectively), even though the difference was not statistically significant (*p *= 0.09).

Multivariate analysis showed that a dominant P-HGP (≤ 50%) was a statistically significant independent favorable prognostic factor for both OS and DFS, as shown in Table 2 (hazard ratio [HR] 2.4, 95% confidence interval [CI] 1.3–4.5, *p *= 0.0069; and HR 1.9, 95% CI 1.1–3.3, *p *= 0.0189, respectively).

**Table 2:** Overall survival and disease-free survival on multivariate analysis (Cox regression model)

	Variable	OS		DFS	
		HR (95% CI)	*p*-Value	HR (95% CI)	*p*-Value
Total population (76)	P-HGP ≤50%	2.4 (1.3–4.5)	**0.0069**	1.9 (1.1–3.3)	**0.0189**
	PCI ≤6	0.3 (0.2–0.7)	**0.0034**	0.2 (0.1–0.4)	**<0.0001**
	Absence of liver metastasis	0.4 (0.2–0.8)	**0.0051**	0.4 (0.2–0.8)	**0.0067**
	Age ≤59 years	0.9 (0.5–1.7)	0.7673	0.4 (0.2–0.9)	**0.0154**
Patients without liver metastasis (40)	P-HGP ≤50%	5.3 (1.8–15.2)	**0.002**	2.6 (1.1–5.7)	**0.0238**
	PCI ≤6	0.3 (0.1–0.8)	**0.0131**	0.3 (0.1–0.6)	**0.002**
	Age ≤59 years	0.9 (0.3–2.4)	0.8534	0.7 (0.3–1.6)	0.3646

Bold values indicate statistically significant results (*p* ≤ 0.05)

*CI* confidence interval, *DFS* disease-free survival, *HR* hazard ratio, *OS* overall survival, *P-HGP* pushing histological growth pattern, *PCI* Peritoneal Cancer Index

Moreover, after exclusion of patients with LM, P-HGP ≤ 50% remained significant for OS and DFS (*p *= 0.002 and 0.0238, respectively).

### Association Between Neoadjuvant Chemotherapy and HGP

Of the 76 patients included in the study, 26 had received NAC before CRS and 50 had not. Among those who did not receive NAC, 26 (52%) had a dominant P-HGP and 24 (48%) had a dominant I-HGP. In the subgroup of patients who had received NAC, 11 (42%) had a dominant P-HGP and 15 (58%) had a dominant I-HGP. Dominant P-HGP was observed in a greater proportion of patients who did not receive NAC than in those who did; however, the association between NAC and HGP type was not statistically significant.

## Discussion

Recently, we reported two HGPs in PMCRC with different prognoses in selected patients (PCI ≤ 6, absence of NAC-treated patients). We conducted this study to further verify and validate the reproducibility and prognostic impact of these patterns in a larger population undergoing curative-intent CRS ± HIPEC, including patients with any PCI, and those treated with NAC. Using the same histopathology evaluation protocol, we observed the two previously described HGPs with the same pathological characteristics: the ‘pushing type’, characterized by an absence of focal penetration of tumor cells into the surrounding tissue; and the ‘infiltrating type’, characterized by focal penetration of tumor cells into the surrounding tissue.

In addition, the same as in our previous study,^18^ pathologists’ readings were almost identical. Moreover, very little difference was observed between the HGP of different nodules from the same patient. Therefore, in theory, it is safe to say that a few peritoneal nodules can be representative of all the PMs. This is very important, as during exploratory laparoscopy it is not feasible to sample the whole peritoneum, therefore sampling a few nodules can serve to determine the HGP status of the PM.

The prognostic impact of HGP on DFS and OS was confirmed in our study, patients with a dominant pushing-HGP > 50% had better DFS (12 months) and OS (60 months) than patients with a non-dominant P-HGP (8 and 39 months, respectively). While the cut-off for OS was P-HGP > 60% in the previous series,^18^ likely due to the small sample size included, the statistically significant cut-offs in this study were 50% for both DFS and OS, adjusting to the small discrepancy through the larger population included. This result correlates well with the definition of dominant pattern in the literature on LM.^20^

In the initial study, patients who received NAC after a diagnosis of PM were excluded because it had been reported previously that preoperative chemotherapy alters the HGP of LMCRC.^19,21^ In this study, we included patients treated with NAC. Surprisingly, and in contrast to HGP in LM, we did not observe a correlation between NAC and the type of PM HGP. However, our analysis was based on only 76 patients, of whom 26 had received NAC, therefore the independence of HGP prevalence from NAC in PMCRC needs to be further explored in a larger cohort.

Given that we have confirmed the observation and prognostic impact of two distinct HGPs in a large PMCRC population, we are more convinced that these patterns could represent a novel prognostic histopathological biomarker that can be used in association with previously known factors to better assess the eligibility of PMCRC patients for CRS ± HIPEC. Since a diagnostic laparoscopy is often performed to confirm a diagnosis of PMCRC and its extent before CRS, we can easily sample PMs to evaluate their HGP, and the type of PM HGP could enter into the decision-making process regarding whether to perform CRS or not.

The limitations of our study are its retrospective and monocentric design, including a limited number of patients and limited number of nodules for each patient. These observations should be further confirmed in large multicentric cohorts. Discussions with other referral centers and peritoneal carcinomatosis teams are already in progress for future collaborations and validation of our findings. Other future studies and perspectives should include a correlation between LM HGP and PM HGP, and between PM HGP and commonly known and used disease tumor markers.

## Conclusion

PMCRC can express two distinct types of HGP independently of the extent of the carcinomatosis and NAC. Patients with a dominant P-HGP treated by CRS ± HIPEC have a more favorable prognosis in terms of both DFS and OS.

## Data Availability

Research data supporting this publication are available upon the Editor’s request.
